# TiO_2_ Nanoparticles/Nanotubes for Efficient Light Harvesting in Perovskite Solar Cells

**DOI:** 10.3390/nano9030326

**Published:** 2019-03-01

**Authors:** Hwa-Young Yang, Won-Yeop Rho, Seul Ki Lee, Sang Hoon Kim, Yoon-Bong Hahn

**Affiliations:** 1 School of Semiconductor and Chemical Engineering, Solar Energy Research Center, Chonbuk National University, Jeollabuk-do 54896, Korea; shot7108@gmail.com (H.-Y.Y.); kalyber@naver.com (S.K.L.); semikim77@gmail.com (S.H.K.); 2 School of International Engineering and Science, Chonbuk National University, 567 Baekje-daero, Deokjin-gu, Jeonju-si, Jeollabuk-do 54896, Korea; rho7272@jbnu.ac.kr

**Keywords:** perovskite solar cells, TiO_2_ nanotube arrays, anodization, light harvesting

## Abstract

To enhance the light harvesting capability of perovskite solar cells (PSCs), TiO_2_ nanoparticles/nanotubes (TNNs) were incorporated into the active layer of PSCs. The TNN-containing cells showed a substantial increase in photocurrent density (*J_SC_*), from 23.9 mA/cm^2^ without nanotubes to 25.5 mA/cm^2^, suggesting that the TiO_2_ nanotubes enhanced the charge conduction and harvested more sunlight, which was attributed to the Mie scattering effect. Compared to the power conversion efficiency (PCE) of TiO_2_ nanoparticles in the active layer (14.16%), the TNN-containing cells with optimal loading of 9 wt % TiO_2_ nanotubes showed a high PCE of 15.34%.

## 1. Introduction

Since Miyasaka introduced organometal halide perovskites into solar cells in 2009, perovskite solar cells (PSCs) have been a hot research topic in next-generation solar cells due to their capacity for absorption across a wide range of visible light [1,2,3,4,5,6,7,8,9,10,11,12,13]. Perovskite consists of Pb, methylammonium, and halide in a cubic structure with a tolerance factor (*t*) of 0.87–1.0 [6,14,15,16,17,18]. The structure of a perovskite solar cell consists of transparent conducting oxide (TCO), a compact layer, electron transport materials (ETMs), perovskite, hole-transport materials (HTMs), and a top electrode [19,20,21,22,23,24,25]. To improve energy conversion efficiency, zero-dimensional TiO_2_ nanoparticles have been used as ETMs in perovskite solar cells and dye-sensitized solar cells due to the presence of a large band gap semiconductor and a large surface area [9,26,27,28,29]. Recently, perovskite stability, Au-free back electrodes, Pb-free sensitizers, various additives, additive-free HTMs, large-area modules, and Indium tin oxide (ITO)/Fluorine-doped tin oxide (FTO)-free devices have been studied substantially [1,3,5,30,31,32,33,34,35]. The structure of perovskite is ABX_3_, which is very weak under humidity, heat, light, and oxygen. To enhance resistance to moisture [30,36], lead (Pb) is replaced by tin (Sn) for a Pb-free sensitizer [37]. To ensure device performance, additives are generally required in HTMs. However, the organic additives reduce the stability of PSCs, and thus they are replaced by metal oxides for long-term stability. Furthermore, the electron transport of zero-dimensional TiO_2_ nanoparticles is no better than that of higher-dimensional TiO_2_ nanostructures. It has been reported that TiO_2_ nanotubes are useful in dye-sensitized solar cells to improve electron transport or to enhance light harvesting because they are three-dimensional nanostructures [38,39,40,41,42]. In general, TiO_2_ nanotubes are prepared using hydrothermal or electrochemical methods. In hydrothermal methods, many individual TiO_2_ nanotubes are synthesized. However, in the electrochemical method, also called anodization, highly ordered and well-aligned TiO_2_ nanotubes are synthesized. The size, length, thickness, and width of the TiO_2_ nanotubes can be easily controlled on micron and nanometer scales as a function of reaction time, voltage, and concentration of electrolytes [43,44,45,46,47,48,49,50].

There are two light scattering theories from Rayleigh and Mie. Rayleigh scattering theory is applicable to small-sized particles, and Mie scattering theory, proposed by German physicist Gustav Mie, is applicable to large-sized particles. According to the Rayleigh theory, scattering by TiO_2_ nanoparticles (20–30 nm) in the active layer is very weak. However, according to Mie scattering theory, submicrometer-sized TiO_2_ nanoparticles are used effectively in the light scattering layer [51,52,53,54]. Individual TiO_2_ nanotubes synthesized by a hydrothermal method are not suitable for light scattering, but the flakes of TiO_2_ nanotubes synthesized by anodization are suitable for light scattering according to the Mie scattering theory. In this study, TiO_2_ nanotube arrays were prepared by anodization and applied for perovskite solar cells (PSCs). To enhance the light harvesting capability of PSCs, TiO_2_ nanoparticles/nanotubes (TNNs) were incorporated into the active layer of PSCs. The TNN-containing cells showed a substantial increase in photocurrent density (*J_SC_*), suggesting that the TiO_2_ nanotubes enhanced the charge conduction and harvested more sunlight, which is attributed to the Mie scattering effect.

## 2. Materials and Methods

### 2.1. Synthesis of TiO_2_ Nanotube Arrays

A Ti plate was cleaned with water, ethanol, and acetone several times using a sonicator and was then dried. The anodization of the Ti plates was carried out in an electrolyte composed of 0.8 wt % NH_4_F and 2 vol % H_2_O in ethylene glycol at 25 °C at a constant applied voltage of 60 V. The TiO_2_ nanotube arrays on the Ti plates were sintered at 500 °C for 1 h under ambient conditions to improve their crystallinity. To obtain free-standing TiO_2_ nanotube arrays, a secondary anodization was carried out at a constant applied voltage of 30 V DC for 10 min, and then the Ti plates were immersed in a 10% H_2_O_2_ solution for 20 min.

### 2.2. Synthesis of Methylammonium Iodide and Preparation of Perovskite Solution

Methylammonium iodide (MAI) was synthesized using a methylamine solution (33 wt % in ethanol) and hydroiodic acid (57 wt % in water). First, methylamine was stirred using a dropwise addition of hydroiodic acid in an ice bath for 2 h. The solvent was evaporated by a rotary evaporator, and then the mixture was solvated in ethanol. After recrystallization with diethyl ether, the white solid was precipitated and then dried under a vacuum for 24 h. The perovskite solution was prepared with MAI and lead (II) chloride (99.999%, Sigma-Aldrich, St. Louis, MO, USA) at a 3:1 molar ratio, 45 wt %, in *N,N*-dimethylformamide (DMF).

### 2.3. Fabrication of the Perovskite Solar Cells

**Figure 1:**
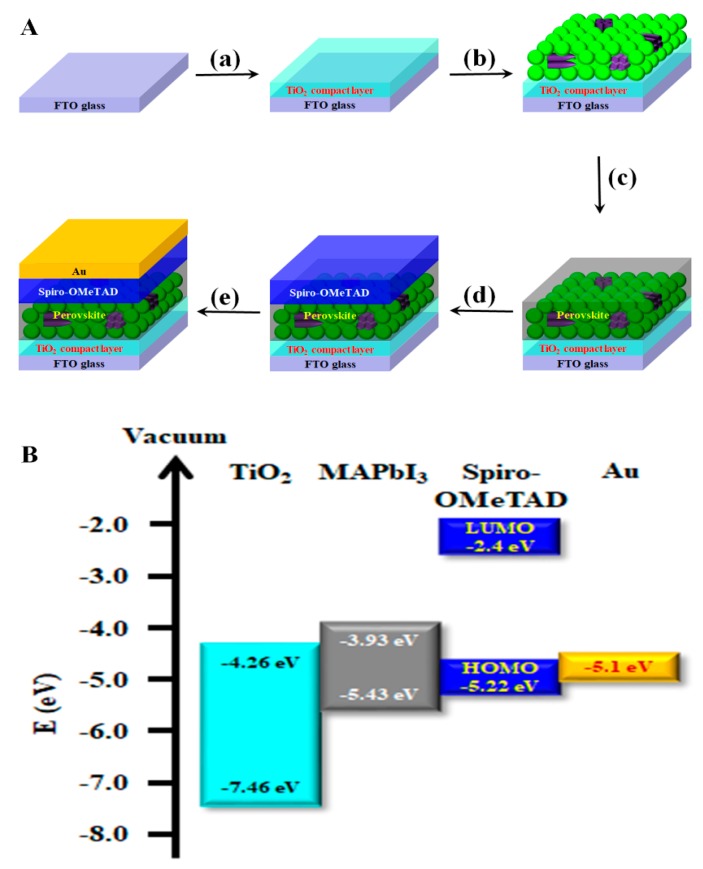
(**A**) Schematic illustration of fabrication processes of perovskite solar cells with TiO_2_ films, including TiO_2_ nanoparticles and flakes of TiO_2_ nanotubes, and (**B**) an energy band diagram of the device.

Figure 1A shows the schematic fabrication process of the PSCs incorporating TiO_2_ nanotubes into the active layer. First, the compact layer of TiO_2_ was prepared by spin-coating 12 wt % titanium diisopropoxide bis(acetylacetonate) in butanol on the fluorine-doped tin oxide (FTO) substrate (a). The flakes of TiO_2_ nanotubes mixed with TiO_2_ nanoparticles were spin-coated on the compact TiO_2_ layer to form a TiO_2_ nanoparticle/nanotube (TNN) film as an electron acceptor, electron transport, and light harvesting layer (b). The TiO_2_ nanoparticles were prepared using TiO_2_ paste (Ti-Nanoxide T/SP, solaronix) diluted in anhydrous ethanol. The TNN films were annealed at 500 °C for 1 h to improve crystallinity. The perovskite film was then coated onto the TNN film by a hot-casting technique at 90 °C, followed by annealing at 130 °C for 1 h (c). The hole-conductor layer of spiro-OMeTAD was formed on the active layer (d). The hole transport material was prepared with 73.52 mg of spiro-OMeTAD (60 mMol), 17 μL of Li [bis-(trifluoromethanesulfonyl) imide] (Li-TFSI) solution (57.42 mg of Li-TFSI in 1 mL of acetonitrile), and 36.22 μL of 4-*tert*-bytylpyridine (500 mMol) in 1 mL of chlorobenzene. Finally, the top electrode of gold was formed by thermal evaporation (e). The energy band diagram of the perovskite solar cells with TiO_2_ inclusion is as shown in Figure 1B. The band gap of the TNN film was similar to that of mesoporous TiO_2_ film. The main role of the TNN film was to enhance light harvesting via the TiO_2_ nanoparticles and/or nanotubes.

### 2.4. Analysis

The photocurrent density-voltage (*J-V*) plots of the perovskite solar cells were measured using a Keithley series 2400 source meter (Tektronix, Beaverton, Portland, OR, USA) under AM1.5 illumination (100 mW/cm^2^) provided by a 150-W Xenon solar simulator (Oriel Corp., model 91160A, Irvine, CA, USA). To examine the crystallinity of TiO_2_ nanotubes, X-ray diffraction (XRD) analysis was performed with a Rigaku D/max-2500 (Rigaku Corp., Tokyo, Japan) using Cu Kα radiation. The absorption properties of the films were examined with ultraviolet-visible (UV-vis) spectroscopy using a JASCO(V-730) spectrometer (JSACO, Easton, MD, USA). The incident photon-to-current efficiency (IPCE) of the devices was measured using a monochromator coupled with a lock-in amplifier and a 500-W Xenon lamp (PV Measurements Inc., Model QEX7, Washington, DC, USA).

## 3. Results and Discussion

**Figure 2:**
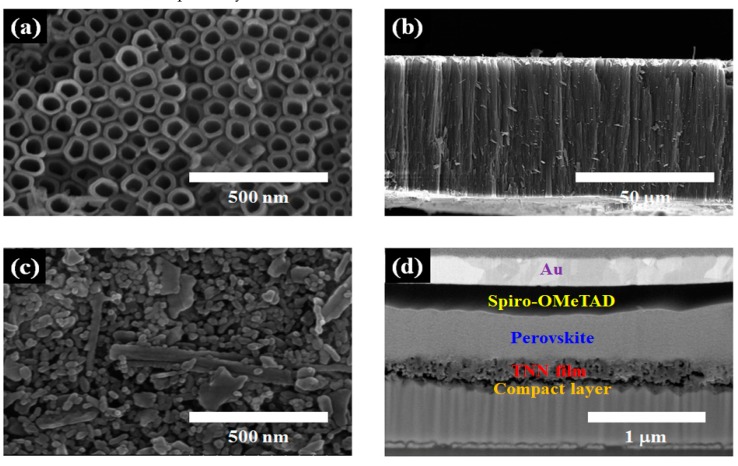
Field emission scanning electron microscopy (FE-SEM) images: (**a**) Top view and (**b**) cross-sectional view of TiO_2_ nanotube arrays; (**c**) top view of TiO_2_ nanoparticle/nanotube (TNN) film; (**d**) cross-sectional view of the perovskite solar cell structure, obtained via focused ion beam (FIB).

Figure 2 shows field emission scanning electron microscopy (FE-SEM) images of the TiO_2_ nanotube arrays (Figure 2a,b) and the TNN film (Figure 2c,d). Pore diameter, wall thickness, interpore distance, and length of the TiO_2_ nanotubes were approximately 100 nm, 20 nm, 200 nm, and 50 μm, respectively. Figure 2c exhibits the top view of the TiO_2_ nanoparticle/nanotube (TNN) film formed on the FTO glass, which shows some flakes of TiO_2_ nanotubes incorporated into the film. Figure 2d shows a cross-sectional SEM image of the perovskite solar cell configuration, obtained using a focused ion beam (FIB) method. The thickness of the TiO_2_ compact layer, TNN layer, perovskite layer, hole-transport layer, and Au electrode were approximately 50 nm, 230 nm, 450 nm, 250 nm, and 260 nm, respectively.

**Figure 3:**
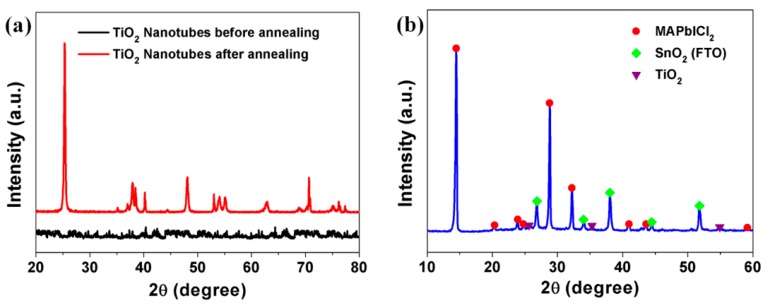
XRD patterns of (**a**) TiO_2_ nanotube film before and after annealing and (**b**) perovskite film on TiO_2_/fluorine-doped tin oxide (FTO).

Figure 3 shows the X-ray diffraction (XRD) patterns of the TiO_2_ nanotube arrays before and after thermal annealing at 500 °C for 1 h (Figure 3a) and the perovskite film on TiO_2_/FTO (Figure 3b). The as-prepared TiO_2_ nanotube arrays by anodization (black) were amorphous, but the annealed nanotubes (red) had crystalline phases of (101), (004), (200), (105), (211), and (118) at 2θ values of 25°, 38°, 48°, 53°, 55°, and 62°, respectively. A dominant peak at 25°, i.e., the (101) peak, was attributed to an anatase crystal phase (Figure 3a). The crystallinity and purity of the perovskite films were confirmed with strong peaks of (110) and (220) at 2θ values of 14° and 28°, respectively, without a PbCl_2_ peak (Figure 3b), indicating that the tetragonal conformation of the perovskite structure was formed.

**Figure 4:**
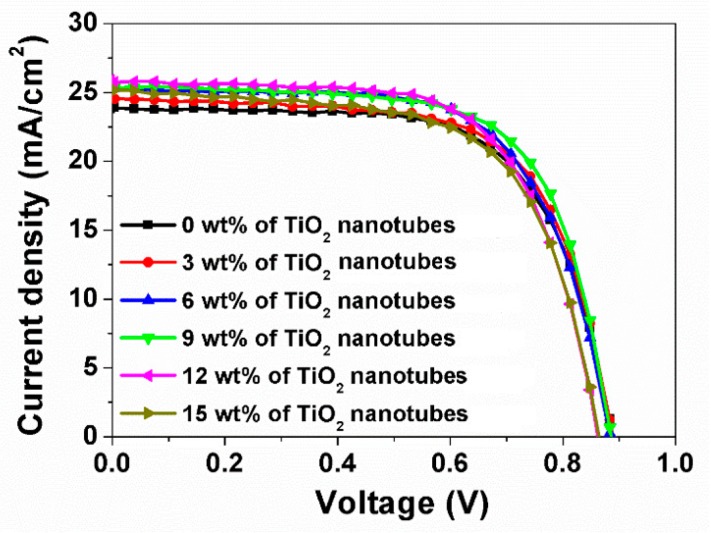
Current density-Voltage (*I-V*) curves of perovskite solar cells with 0, 3, 6, 9, 12, and 15 wt % of TiO_2_ nanotube arrays in a TiO_2_ composite film.

Figure 4 shows photocurrent density-voltage (*J-V*) curves of the TNN-based perovskite solar cells as a function of weight percentage of TiO_2_ nanotubes in the TNN-containing active layer. The corresponding photovoltaic parameters of the cells are summarized in Table 1. Compared to the TiO_2_ nanoparticles only, the TNN-containing cells showed better performances with higher values of short circuit current density (*J_SC_*), fill factor (*FF*), and power conversion efficiency (*η*). The optimal content of TiO_2_ nanotubes in TNNs was 9 wt %, resulting in 0.886 V of *V_oc_*, 25.5 mA/cm^2^ of *J_SC_*, 67.9% of *FF*, and 15.335% of *η*. More interestingly, the TNN-containing cells showed a substantial increase in *J_SC_*, from 23.9 mA/cm^2^ without nanotubes to 25.5 mA/cm^2^ with 9 wt % nanotubes, suggesting that the micronmeter-sized TiO_2_ nanotubes enhanced the charge carrier generation by harvesting more sunlight, probably attributed to the Mie scattering effect. To check the stability of TNN-based PSCs, we exposed the devices without encapsulation to an Ar environment at room temperature. Figure S1 (Supplementary Materials) shows *V_oc_*, *J_SC_*, *FF*, and *η* from the *J-V* test results for 80 days. Overall, the photovoltaic parameters of the devices were stabilized after 40 days and sustained their stability over 80 days, while retaining 95%–99% of their original values. To evaluate the reproducibility of the TNN-based PSCs, we fabricated 20 devices and measured device performance. Figure S2 shows the histogram of the power conversion efficiency distribution for 20 devices, apparently presenting the best performance of the PSCs with 9 wt % of TiO_2_ nanotubes, i.e., *η* = 14%–15%.

**Figure 5:**
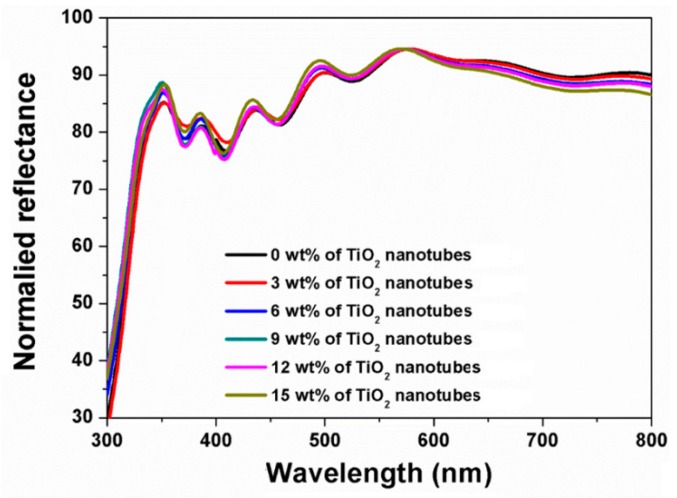
Normalized reflectance spectra of TiO_2_ composite films with 0, 3, 6, 9, 12, and 15 wt % of TiO_2_ nanotubes.

To confirm the light harvesting effect of TiO_2_ nanotube flakes in the active layer, the reflectance spectra of the TNN films and the IPCE spectra of the TNN-containing perovskite solar cells were measured. Figure 5 shows the normalized reflectance spectra in the TNN films as a function of weight percentage of TiO_2_ nanotubes. As the content of TiO_2_ nanotubes increased, the TNN films showed a similar trend of reflectance, but with some variation in intensity. At wavelengths greater than 570 nm, the intensity of the reflectance decreased with the amount of the TiO_2_ nanotubes, indicating more light harvesting by TiO_2_ nanotubes at longer wavelengths (i.e., 570–800 nm), thus enhancing charge carrier generation in the active layer.

**Figure 6:**
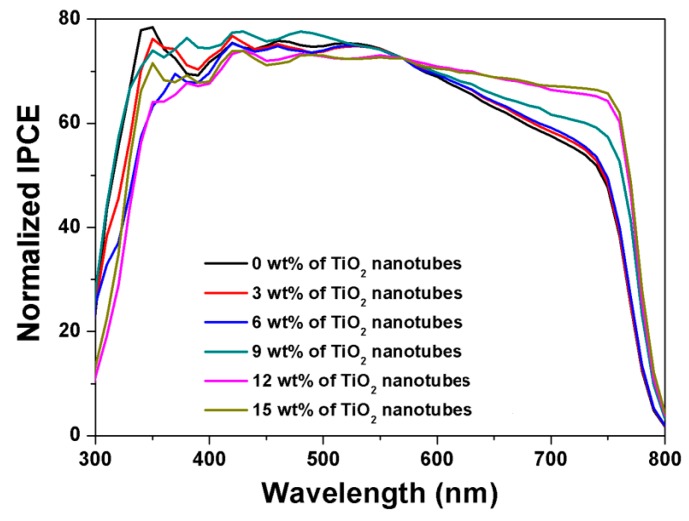
Normalized incident photon-to-current efficiency (IPCE) spectra of perovskite solar cells with 0, 3, 6, 9, 12, and 15 wt % of TiO_2_ nanotubes in the active layer.

Figure 6 shows the normalized IPCE spectra from TNN-containing perovskite solar cells with varying content of the TiO_2_ nanotubes from 0 to 15 wt %. With increasing amounts of TiO_2_ nanotubes, the photon-to-current efficiency increased substantially at wavelengths greater than 570 nm, which was in good agreement with Figure 4 and Figure 5, attributed to more light harvesting due to a scattering effect by nanotubes and more generation of charge carriers in the active layer. However, it is worthwhile to note that the incorporation of nanotubes of more than 12 wt % increased the recombination rate and thus decreased the photocurrent density that affected *FF* and *V_oc_* (also see Table 1). Generally, it is known that according to Rayleigh theory, scattering by TiO_2_ nanoparticles of 20–30 nm is very weak [51,54]. The pore diameter of TiO_2_ nanotubes was 100 nm (Figure 2a), and the length of nanotubes in the TNN film was in the range of 300–1000 nm in size (Figure 2c): Thus, the enhancement of light harvesting with the TNN films was probably attributed to the Mie scattering effect. In addition, as the TNN films were mesoporous, perovskite solution easily penetrated into the TNN film layer. Thus, it is believed that scattering was toward the perovskite as well as in the direction of the tubes.

## 4. Conclusions

Perovskite solar cells were fabricated with the inclusion of TiO_2_ nanoparticles/nanotubes as light harvesting materials and were characterized in terms of the normalized reflectance and IPCE. The TiO_2_ nanoparticle/nanotube-based cells harvested more sunlight with the content of the nanotubes (attributed to the Mie scattering effect) and thus enhanced the carrier charge generation and conduction. However, a large amount of TiO_2_ nanotubes did not result in improved energy conversion efficiency because of high levels of recombination and low electron density in the active layer. With the optimal content of the TiO_2_ nanotubes (i.e., 9 wt %), the devices showed high photocurrent density and a power conversion efficiency of 15.34%. The TNN-based PSCs also showed good stability, retaining 95–99% of their initial photovoltaic parameter values. The obtained results could be applicable to different types of solar cells and photocatalysts and water splitting technology for hydrogen generation.

## Figures and Tables

**Table 1:** Photovoltaic properties of perovskite solar cells with 0, 3, 6, 9, 12, and 15 wt % of TiO_2_ nanotube arrays in a TiO_2_ composite film.

	*V_oc_* (V)	*J_SC_* (mA/cm^2^)	*FF* (%)	*η* (%)
0 wt %	0.888	23.908	66.694	14.162
3 wt %	0.888	24.705	66.018	14.489
6 wt %	0.884	25.307	66.330	14.834
9 wt %	0.886	25.500	67.906	15.335
12 wt %	0.863	25.888	65.744	14.684
15 wt %	0.865	25.223	63.996	13.960

## Supplementary Materials

The following are available online at https://www.mdpi.com/2079-4991/9/3/326/s1, Figure S1: The stability of perovskite solar cells with 9wt% of TiO_2_ nanotubes in a TiO_2_ composite film, Figure S2: The histograms of PCE with 0, 9 and 15wt% of TiO_2_ nanotubes in active layer.
