# Supplementary material for: TiO2 Nanoparticles/Nanotubes for Efficient Light Harvesting in Perovskite Solar Cells
Source: Nanomaterials (Basel). 2019 Mar 1;9(3):326. doi: 10.3390/nano9030326 (PMC6473427; doi:10.3390/nano9030326)
Supplement: Supplementary file 1 [file nanomaterials-09-00326-s001.pdf]

Supplementary Materials

TiO_2 Nanoparticles/Nanotubes for Efficient Light Harvesting in Perovskite Solar Cells

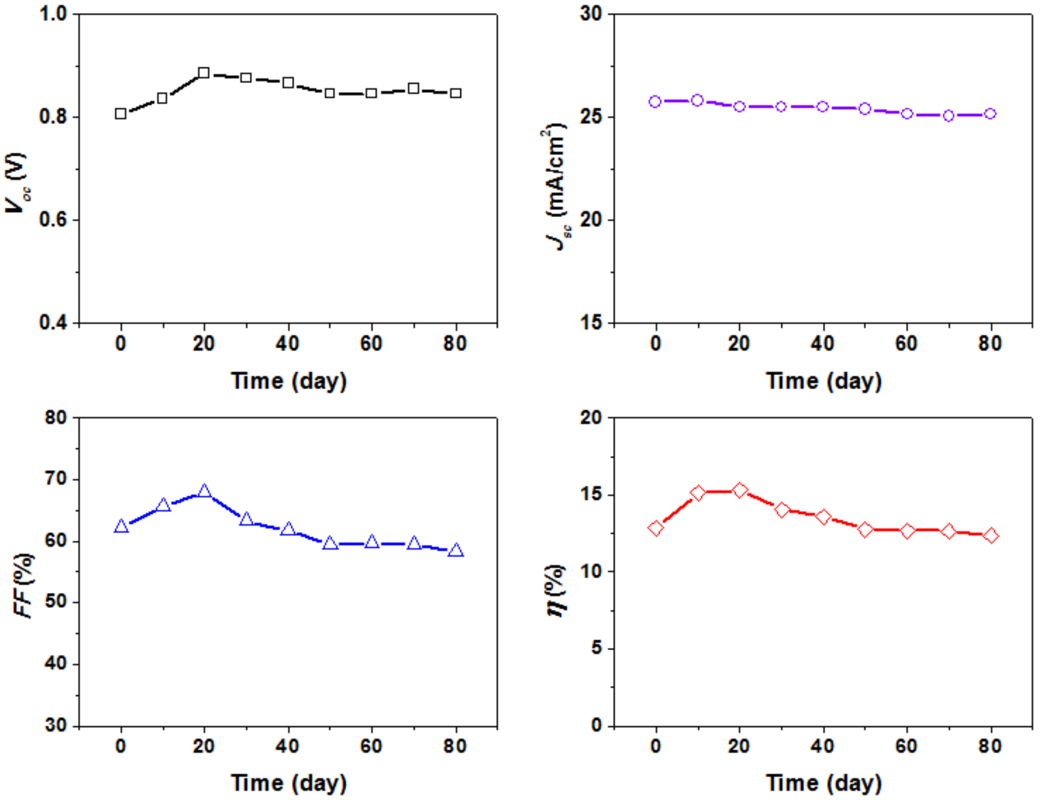

Figure 1. The stability of perovskite solar cells with 9 wt % of TiO_2 nanotubes in a TiO_2 composite film: **(a)** V_{oc}, **(b)** J_{sc}, **(c)** *FF*, and **(d)** efficiency (η).

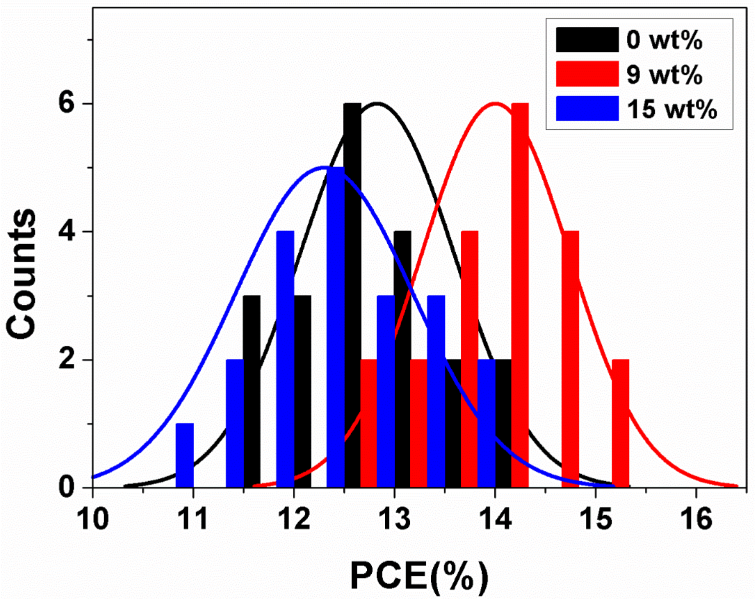

Figure S2. The histograms of power conversion efficiency (PCE) with 0 wt % (black), 9 wt % (red), and 15 wt % (blue) of TiO_2 nanotubes in the active layer.
